# Structural, Electronic, Mechanical, and Thermodynamic Properties of Na Deintercalation from Olivine NaMnPO_4_: First-Principles Study

**DOI:** 10.3390/ma15155280

**Published:** 2022-07-30

**Authors:** Ratshilumela S. Dima, Prettier M. Maleka, Nnditshedzeni E. Maluta, Rapela R. Maphanga

**Affiliations:** 1 Next Generation Enterprises and Institution Cluster, Council for Scientific and Industrial Research, P.O. Box 395, Pretoria 0001, South Africa; sdima@csir.co.za (R.S.D.); pmaleka@csir.co.za (P.M.M.); rmaphanga@csir.co.za (R.R.M.); 2 Department of Physics, University of Venda, P/Bag X 5050, Thohoyandou 0950, South Africa; 3 National Institute for Theoretical and Computational Sciences (NITheCS), Gauteng 2000, South Africa

**Keywords:** elastic properties, first-principle calculations, sodium-ion batteries, NaMnPO_4_, olivine

## Abstract

The impact of Na atom deintercalation on olivine NaMnPO_4_ was investigated in a first-principle study for prospective use as cathode materials in Na-ion batteries. Within the generalized gradient approximation functional with Hubbard (U) correction, we used the plane-wave pseudopotential approach. The calculated equilibrium lattice constants are within 5% of the experimental data. The difference in equilibrium cell volumes for all deintercalated phases was only 6%, showing that NaMPO_4_ is structurally more stable. The predicted voltage window was found to be between 3.997 and 3.848 V. The Na_1_MnPO_4_ and MnPO_4_ structures are likely to be semiconductors, but the Na_0_._75_MnPO_4_, Na_0_._5_MnPO_4_, and Na_0_._25_MnPO_4_ structures are likely to be metallic. Furthermore, all independent elastic constants for Na_x_MPO_4_ structures were shown to meet the mechanical stability requirement of the orthorhombic lattice system.

## 1. Introduction

Sodium-ion batteries (SIBs) are considered a feasible alternative to lithium-ion batteries (LIBs) as they have the potential to be much less expensive, safer, and ecologically friendly [1]. Based on the promising results reported in previous studies [1,2,3], several discoveries revealed that there is a significant opportunity for materials design and development in low-temperature Na-ion battery chemistries. During charging and discharging, Na^+^ ions shuttle between the positive and negative electrodes in Na-ion batteries, similarly to Li-ion batteries, with an electrolyte acting as a transport medium for those ions. A critical stage in the deployment of large-scale SIB applications is the search for suitable electrode materials that meet long-cycle stability criteria and can store and transport huge amounts of energy quickly. Several phases that allow Li-ion (de) intercalation have been explored as Na^+^ hosts, including layered metal oxides Na_x_MO_2_ [4,5,6] and polyanionic compounds [7,8], due to their similar intercalation chemistries. However, using standard Na equivalents, performance comparable to the high-rate capability and cycling stability of LIB electrodes is difficult to achieve [9]. The main reason for this is that Na^+^ has a much larger ionic radius (r = 0.102 nm) than Li+ (r = 0.076 nm), resulting in slower ion mobility and larger volume changes in its host structure [10]. In layered oxides produced through cubic closed-packed oxide arrays, Na^+^ transport is slow, and Na ion deintercalation causes complex phase transitions, resulting in rapid electrode degradation [6].

**Figure 1:**
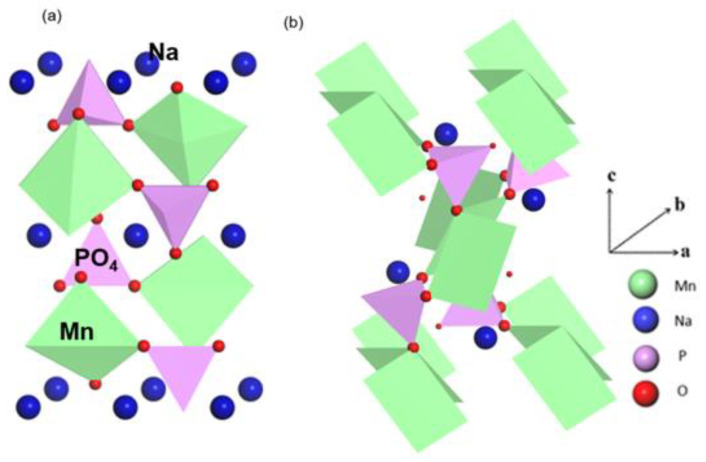
Schematic crystallographic structures of (**a**) olivine and (**b**) maricite NaMnPO_4_.

Lithium transition metal phosphates of olivine type, such as LiFePO_4_, have increased cycle and thermal stability as cathode materials compared to systems based on lithium oxide [11,12,13]. Furthermore, although the volumetric discrepancy between the olivine structured LiFePO_4_ and the de-lithiated FePO_4_ is 6.9%, the mismatch between the Na analogs is 17.58% [14]. During cycling, such a large volume shift has a detrimental impact on structural stability. In this setting, novel Na host materials that promote viable Na^+^ (de)-intercalation while requiring minimal volume change are crucial. The expense of electrode materials designed for SIBs is another important concern. Despite the fact that Na ions are a low-cost charge carrier, many reported cathode materials still rely on redox centers that are scarce and/or require hazardous transition metal components such as cobalt [4,15], nickel [15], vanadium [16] and chromium [6]. As a result, the cost and sustainability advantages of SIBs are considerably reduced. Large-scale applications necessitate the development of electrode materials based on Earth’s abundance of transition metals, with Fe and Mn being the most common transition metals in the Earth’s crust. Inspired by the LiMnPO_4_ structure, NaMnPO_4_ has recently attracted the attention of several researchers as a cathode material for SIBs. NaMnPO_4_ has two structural variations compared to LiMnPO_4_ namely, maricite and olivine type [17,18]. The phosphate group framework is the same in both configurations (space group Pnma) (Figure 1). The difference arises from the fact that in the olivine structure (Figure 1a), Na^+^ and Mn^2+^ ions prefer to occupy two octahedral positions, but in the maricite-type structure, the opposite is true (Figure 1b). In the olivine structure, the Na^+^ containing octahedral sites share edges and form zigzag chains along the b-axis [17]. 

Compared to olivine, maricite has been found to be the most thermodynamically stable phase. The maricite phase is 0.016 eV/formula unit more stable than the olivine phase [19,20]. This material is unlikely to be used in the construction of sodium ion batteries due to the edge-sharing MnO_6_ octahedrons sharing edges and the lack of cationic channels for Na diffusion. In this work, the structural, thermodynamic, electrical, and mechanical properties of olivine NaMnPO_4_ structures are examined to determine their stability and competency as cathode materials. Formation energies, electrical densities of states (DOS), and elastic constants will be computed to model the stability trend at 0 K.

## 2. Computational Method

The Vienna ab initio simulation package (VASP) [21] program was used for all the calculation, in the projector augmented wave (PAW) pseudopotential, which is based on density functional theory (DFT) [22]. A spin-polarized generalized gradient approximation (GGA) was used to solve the Kohn–Sham equations with the Perdew–Burke–Ernzerhof functional (PBE) exchange–correlation functional, with plane-wave pseudopotential [23]. The orbitals Na (3s), Mn (3d, 4s), O (2s, 2p) and P (3p) are studied for their valence states. The Hubbard correction term U (i.e., GGA + U) was also utilized because of the high Coulomb repulsion at site among Mn 3d electrons. We use an effective U value of 5.0 eV for Mn in this study, which was chosen based on previous research [24] and validated by our test calculations. The results presented here are based on the ferromagnetic configuration because the ground-state energies of the molecule determined from other spin configurations are so close. The Monkhorst–Pack scheme was used, with a 5 × 5 × 7 k-point mesh. The cutoff energy for the plane wave function was 560 eV. When the final force on all ions is less than 0.01 eV/Å, the atomic locations and lattice parameters are considered totally relaxed. The elastic properties were calculated by means of Taylor expansion of total energy using a strain of 0.005. Lastly, the densities of states were calculated using Gaussian smearing with a width of 0.05 eV for both spin up and down orientations.

## 3. Results and Discussion

### 3.1. Structural Properties

The calculated structural properties of NaMnPO_4_ are listed in Table 1. The calculated lattice parameters, a, b, and c are within 2% of the deviation from the experimental results, and the volume deviation was found to be within 5% of the deviation from the reported results. Differences between the calculated and experimental values are found to be small, indicating that the applied theoretical method is moderate.

**Figure 2:**
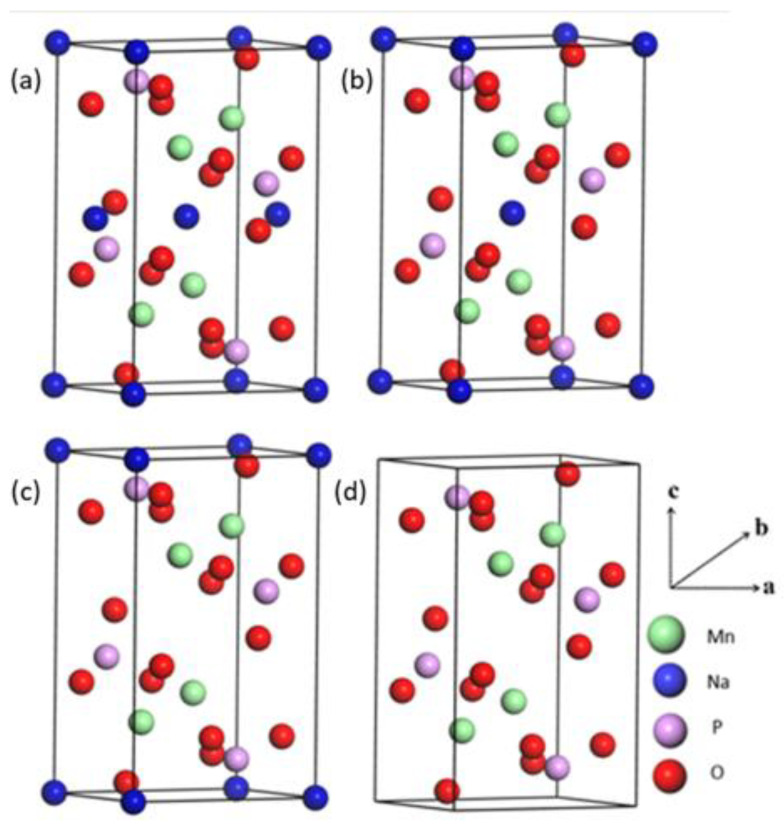
Spin-polarized crystallographic relaxed structures of (**a**) Na_0_._75_MnPO_4_, (**b**) Na_0_._5_MnPO_4_, (**c**) Na_0_._25_MnPO_4_, and (**d**) MnPO_4_ during Na extraction.

The Na extraction process from the NaMnPO_4_ lattice is investigated to simulate the discharge process. During every extraction stage, the Na atom at the original binding site is removed assuming a topotactic approach, which is followed by the relaxation of the resulting structure. The deintercalated Na_x_MnPO_4_ structures are shown in Figure 2. The lattice parameters and changes in cell volume of Na_x_MnPO_4_ systems during Na extraction are listed in Table 2. During Na extraction from Na_x_MnPO_4_ (x = 1, 0.75, 05, 0.25, 0), the lattice parameter and changes in cell volume changes are less than 5%, indicating that structural stability is well maintained throughout the various stages. However, after extraction of the fourth Na atom (x = 0), where the structure is fully deintercalated, the cell volume changed by 5.9%, which was found to be higher than the rest. Note that this volume change is not large enough to cause irreversible destruction of the structure.

The results of a fully deintercalated structure are found to have a lower percentage of deviation than those previously reported by Fang et al. [14]. The study reported on the high-performance olivine NaFePO_4_ microsphere cathode synthesized by the aqueous electrochemical displacement method, with a volumetric mismatch between olivine structured LiFePO_4_ and de-lithiated FePO_4_ of 6.9%, while Na analogs were found to have a larger mismatch of 17.58% [10].

### 3.2. Electronic Properties

**Figure 3:**
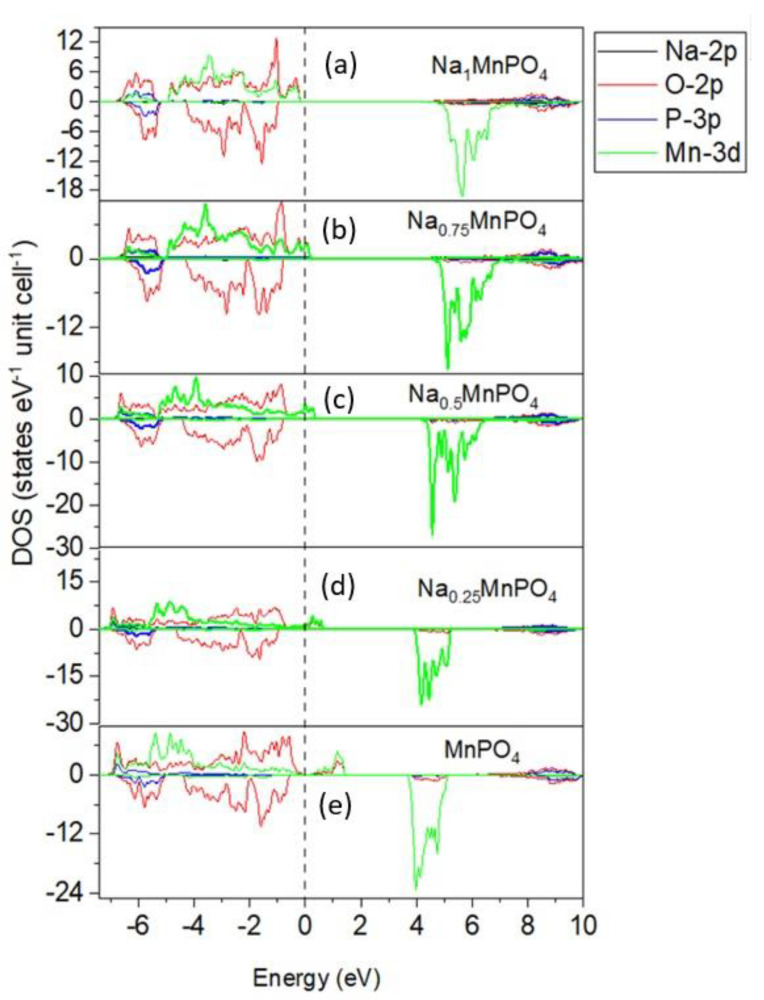
Spin-polarized DOS near Fermi level of Na_x_MnPO_4_ (x = (**a**) 1, (**b**) 0.75, (**c**) 0.5, (**d**) 0.25, (**e**) 0). The Fermi level is set as 0 eV and is shown by the dashed lines. In the DOS curve, the positive and negative values refer to the DOS of the spin-up and spin-down states, respectively.

We calculated the densities of states (DOS) of magnetic Na_x_MPO_4_ systems in the magnetic spin-polarized state to elucidate their electronic conductivity. Total and orbital partial DOS are separated, generating a band gap near the Fermi level separating the conduction band and the conduction band, as shown in Figure 3a. To comprehend the electronic conductivity of materials, concepts of Fermi level and band gaps are required. The Fermi energy is employed as the energy scale’s zero, and the energy band gaps for both spin-up and spin-down states were observed. The states near the Fermi level are mostly Mn 3d and O 2p, with just minor contributions from the Na and P states. Due to the smaller band gap, it can be concluded that the spin-up states are primarily insulators (according to theoretical data [26]), whereas the spin-down states are semiconductors. The partial DOS of deintercalated Na_x_MPO_4_ structures are presented in Figure 3a–e. For the initial structure Na_1_MnPO_4_, the system was found to be a magnetic insulator with a direct band gap of 3.363 eV, as shown in Figure 3a. The valence band (spin-up) maximum is located near (0.00 0.00 0.00), at −0.203 eV with respect to the Fermi level. The conduction band (spin-up) minimum is located near (0.00 0.00 0.00), at 3.161 eV with respect to the Fermi level. The center of the gap is located at 1.479 eV with respect to the Fermi level.

During extraction, the Fermi level is located on the Mn 3d and O 2p bands, as illustrated in Figure 3b-d, except for the MnPO_4_ system in Figure 3e, suggesting that some states of the valence band jump the Fermi level barrier to the conduction band. The energy band gap value and the location of the Fermi level suggest that Na_0_._75_MnPO_4_ in Figure 3b is semi-metallic, resulting in good electrical conductivity in Na-ion batteries. This metallicity increases as the number of Na atoms extracted increases. However, interestingly, a fully deintercalated MnPO_4_ system was found to be a magnetic semiconductor with a direct band gap of 0.194 eV. The maximum valence band (spin-up) is located near (0.00 0.00 0.00), at −0.100 eV with respect to the Fermi level, whereas the conduction band (spin-up) minimum is located near (0.00 0.00 0.00) at 0.094 eV with respect to the Fermi level. The center of the gap is located at −0.003263 eV with respect to the Fermi level. The Fermi energy is used as the zero of the energy scales. Regarding spin-down, it was found that Mn 3d had moved from ≈5.1 to ≈3.7 eV. Generally, it was observed that the partial density of states reveals that the Mn 3d states contribute significantly to both the conduction band and the valence band, whereas the O 2p states contribute more to the valence band. In addition, there are minimal contributions from the Na and P states. It was also noted that oxygen contributed significantly to the upper states of Na_4_MnPO_4_. Thus, most of the charge compensation for the sodium removal was obtained by oxygen oxidation. Further discussion on which reaction occurs after sodium removal would require considering the kinetics and environment, such as temperature and oxygen pressure. Additionally, it was observed that during each deintercalation stage, the magnetic moments of Mn altered, decreasing from 4.5 µ_B_ for Na_4_MnPO_4_ to 3.85 µ_B_ for MnPO_4_. Hence, the total magnetic moments for the cell were found to be decreasing as the number of Na atoms removed increase; these total cell magnetic moments ranged from 20 µ_B_ for Na_4_MnPO_4_ to 16 µ_B_ for MnPO_4_. We used the Bader charge analysis in this study to reveal the change in the electronic population at ionic centers throughout the Na deintercalation process. It was discovered that Mn and O engage in the redox reaction to a lesser extent, contributing an average of 1.757 e and 1.403 e, respectively.

### 3.3. Voltages/Redox Potential and Formation Energy

The energy change of the individual Na atom at every deintercalation stage of the NaMnPO_4_ structure is calculated using analytical expression:

(1) $$\Delta E=E\left(Na_{x-y}MnPO_{4}\right)+yE\left(Na\right)-E\left(Na_{x}MnPO_{4}\right),\;$$

where ΔE is the change in energy of the process that $y\left(Na\right)$ atoms are extracted between the NaMnPO_4_ layers,$\;E\left(Na_{x-y}MnPO_{4}\right)$, $E\left(Na\right)$ and $E\left(Na_{x}MnPO_{4}\right)\;$ are the energy of $\left(Na_{x-y}MnPO_{4}\right)$, Na metal and $Na_{x}MnPO_{4}$ respectively.

Accordingly, the cathode voltage/redox potential is calculated as follows:

(2) U=ΔEye

**Figure 4:**
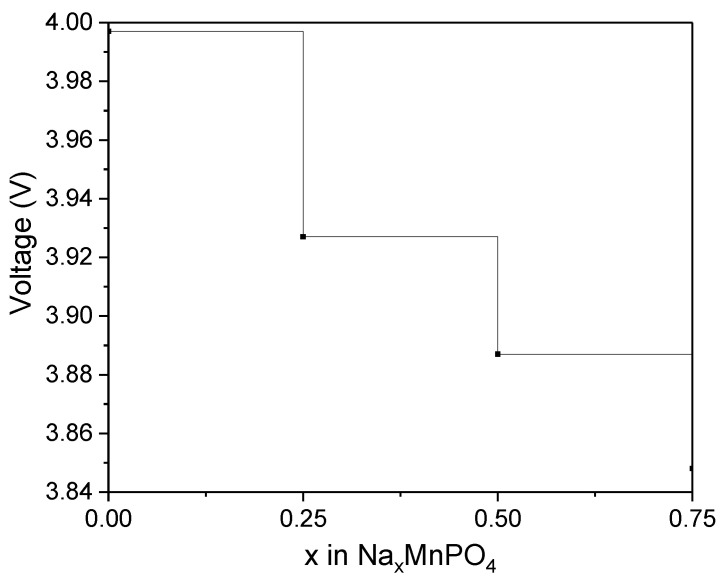
Deintercalation potentials (V) for Na_x_MnPO_4_ (x = 1, 0.75, 0.5, 0.25, 0).

The cathode voltage/redox potential is U, the absolute value of the electron charge is e, and the number of Na atoms extracted is y. Equation (2) was used to calculate the average voltage/average potential at each deintercalation stage, which is shown in Figure 4. The voltage or potential required to remove the Na ions from Na_x_MnPO_4_ ranged between 3.997 and 3.848 V. Moreover, the *Na*_x_Mn_2_O_4_ potentials are consistent with previously calculated data for Mn-based isostructures olivine LiMnPO_4_ [26] and maricite NaMnPO_4_ [19].

To understand the formation of solid solutions of Na removal Na_x_MnPO_4_, the formation energy per unit of formula was calculated using the expression:

(3) $$FE=E\left(Na_{x}MnPO_{4}\right)-x\times E\left(NaMnPO_{4}\right)-\left(1-x\right)E\;\left(MnPO_{4}\right)$$

where E(Na_x_MPO_4_) is the energy of the partially deintercalated material, and E(NaMnPO_4_) and E(MnPO_4_) are the energies of the pristine and totally deintercalated structures, respectively. For Na_1_MnPO_4_, Na_0_._75_MnPO4, Na_0_._5_MnPO_4_, Na0.25MnPO4 and MnPO_4_, the predicted formation energies are −60.12 eV, −45.291, −30.153, and 0, respectively. With the exception of the last stage, when x = 0, we find that the formation energy of the intercalation phases is negative for all materials. The negative value indicates that these materials are predicted to have a solid solution. 

### 3.4. Mechanical Properties

#### 3.4.1. Elasticity

Fundamental solid-state features, such as the equation of state, interatomic potentials, lattice constants, and phonon spectra, are all connected to elastic properties. They include crucial information about the strength of a material against an externally imposed strain and are used as stability criteria in the study of structural stability modifications of mechanical stability [27,28]. Elastic constants of a material, as defined by the bulk modulus (B), shear modulus (G), Young’s modulus (E), Poisson’s ratio (v) and shear anisotropy factor, describe its response to the external applied strain required to maintain a given deformation and provide useful information about the material’s strength (A). Born [29] was the first to calculate elastic constants. The Born stability requirements are a collection of conditions on the elastic constants (C_ij_) that are linked to a crystal’s internal energy shift in the second order during formation. However, the Born stability ranges were later revealed to be sensitive to the choice of locations. Table 3 shows the elastic constants determined using a Taylor expansion obtained from Equation (4) [30].

(4) Uv,ε=UV0,0+V0∑iτiεiδi+12∑ijCijεiδiεjδj

where $U\left(V_{0},0\right)$ is the unstrained system energy, $V_{0}$ is the equilibrium volume, $\tau_{i}$ is the element in the stress tensor, and $\delta_{i}$ is a Voigt index factor. The nine independent elastic constants (C_ij_) of orthorhombic NaMPO_4_ structures are shown in Table 3. For orthorhombic systems, the Born mechanical stability criteria are [26,27,31,32]:

(5) $$\left(C_{11}+C_{22}-2C_{12}\right)>0,\;\left(C_{11}+C_{33}-2C_{13}\right)>0,\;\left(C_{22}+C_{33}-2C_{23}\right)>0,\;C_{11}+C_{22}+C_{33}+2C_{12}+2C_{13}+2C_{23})>0,\;C_{11}>0,\;C_{11}>0,\;C_{22}>0,\;C_{33}>0,\;C_{44}>0,\;C_{55}>0,C_{66}>0$$

We noted that all stability criteria were met, which shows that the olivine structure’s NaMPO_4_ compounds were mechanically stable. During the extraction phases, the first to third Na extraction phases, all Born mechanical stability criteria were met. However, for the fourth Na extraction, which is a fully deintercalated structure, the C_44_ > 0 criteria were not achieved since C_44_ = −39.20 GPa. This phenomenon shows that NaMnPO_4_ cannot be fully deintercalated and still maintain its stability. This is the first elastic constant recorded in the Na_x_MnPO_4_ system when Na was removed.

Using the Voigt–Reuss–Hill method, the macroscopic mechanical parameters of the bulk, shear and Young’s moduli are calculated from the calculated elastic constants [30].

(6) $$B_{V}=\frac{1}{9}\left(C_{11}+C_{22}+C_{33}\right)+\frac{2}{9}\left(C_{12}+C_{13}+C_{23}\right)$$

(7) $$B_{R}=\left[\left(S_{11}+S_{22}+S_{33}\right)+2{\left(S_{12}+S_{13}+S_{23}\right)}^{-1}\right]$$

(8) $$G_{V}=\frac{1}{15}\left(C_{11}+C_{22}+C_{33}-C_{12}-C_{13}-C_{23}\right)+\frac{1}{5}\left(C_{44}+C_{55}+C_{66}\right),$$

(9) $$G_{R}=15{\left[4\left(S_{11}+S_{22}+S_{33}\right)-4\left(S_{12}+S_{13}+S_{23}\right)+3\left(S_{44}+S_{55}+S_{66}\right)\right]}^{-1}$$

(10) $$B_{H}=\frac{1}{2}\left(B_{R}+B_{V}\right)$$

(11) $$G_{H}=\frac{1}{2}\left(G_{R}+G_{V}\right),$$

(12) $$E_{H}=\frac{9B_{H}G_{H}}{G_{H}+3B_{H}},z$$

where B, G and E are the bulk, shear and Young’s moduli, respectively, while V, R and H are the Voigt, Reuss and Hill bounds, respectively, and $S_{ij}$ is the inverse matrix of the elastic constant’s matrix $C_{ij}$, which is given by Ravindran et al. [33]. There are three sets of results in Table 4, which include the bulk, shear and Young’s moduli. Depending on the material’s bulk and Young’s moduli, as well as its shear modulus, its hardness and stiffness may be determined. Pressure-induced volume changes are also taken into account when calculating the bulk modulus; these results were calculated using the VASP-MT package, employing Equation (4). 

The positive bulk, shear, and Young’s moduli of Na_x_MnPO_4_ structures are relatively large, implying hardness, great resistance to volume change, deformation, and stiffness, respectively. The elastic characteristics of the maricite NaMnPO_4_ polymorph were calculated to be higher than those reported here for the olivine phase by Lethole et al. [26]. Furthermore, our calculated C_11_, C_22_, and C_33_ values are much larger than C_44_, C_55_, and C_66_, implying that the materials have strong directional resistance to linear compressions against uniaxial pressures but limited resistance to shear deformations.

Furthermore, we note that B_H_ > G_H_, implying that the shear modulus (G_H_) is the parameter that limits the mechanical stability of the Na_1_MnPO_4_ structures [33]. In addition, the Pugh ductility and brittleness criterion was calculated. Pugh proposed the bulk-to-shear modulus (B/G) ratio for polycrystalline phases, assuming that the shear modulus represents plastic deformation resistance, and the bulk modulus represents fracture resistance. Brittleness is related with a low B/G value, whereas ductility is related with a high B/G value. The value 1.75 is the critical number that distinguishes ductility from brittleness. Because B_H_/G_H_ is greater than 1.75, the structures of the Na_1_MnPO_4_, Na_0_._75_MnPO_4_ and MnPO_4_ structures are ductile, which implies that these materials can bend without deformation, resulting in fewer cracks during battery operation [34]. However, because their predicted B_H_/G_H_ is less than 1.75, Na_0_._5_MnPO_4_ and Na_0_._25_MnPO_4_ are both brittle.

#### 3.4.2. Anisotropy in Elastic Constants

Almost all known crystals have elastically anisotropic behavior, and a proper description of this anisotropic behavior has important implications in both engineering science and crystal physics, as well as in other fields. It is possible to calculate the shear anisotropic factors by calculating the degree of anisotropy in the bonding between atoms that are in various planes. The shear anisotropic factors provide a measure of the degree of anisotropy in the bonding between atoms in different planes. The shear anisotropic factor for the {100} and 〈110〉, 〈010〉 directions is calculated as:

(13) $$A_{1}=\frac{4C_{44}}{C_{11}+C_{33}-2C_{13}}$$

For the ({010}, {001}), the shear planes between the (〈011〉 and 〈010〉 (〈110〉,  〈001〉, respectively) directions are:

(14) $$A_{2}=\frac{4C_{55}}{C_{22}+C_{33}-2C_{23}}$$

(15) $$A_{3}=\frac{4C_{66}}{C_{11}+C_{22}-2C_{12}}$$

Table 5 shows the shear anisotropic factors obtained from our theoretical investigations. Factors A_1_, A_2_, and A_3_ must all be one for an isotropic crystal, whereas any number less than or greater than unity indicates the crystal’s degree of elastic anisotropy. For Na_1_MnPO_4_, the values of A_1_ (1.085), A_2_ (1.145), and A_3_ (1.409) diverge from the unity by 8.50%, 14.50%, and 40.09%, suggesting an isotropic characteristic. During the deintercalation stages, all the materials displayed slight deviations from the unity, whereas A_1_ and A_3_ for MnPO_4_ showed the greatest deviation from the unity, with A_1_ having a negative value. Chung and Buessem [35] developed a new term for non-cubic systems: percent elastic anisotropy [35]. It is a measure of the amount of elastic anisotropy held by the crystal under discussion. The percentage anisotropy in compressibility and shear moduli is described by the equations $A_{B}=B_{V}-B_{R}/B_{V}+B_{R}$ and $A_{G}=G_{V}-G_{R}/G_{V}+G_{R}$, respectively, for compressibility and shear moduli. $B_{R}=B_{V}$ is related with isotropic elastic constants, but a value of 100% is associated with the maximum amount of anisotropy that may be achieved. Deintercalations of Na tend to raise A_B_ and A_G_ values, suggesting an improvement in isotropy and a reduction in microcracks and dislocations in the charge/discharge process, apart from the MnPO_4_ sample. In all stages of deintercalation, the proportion of shear modulus anisotropy A_G_ is lower than the percentage of bulk modulus anisotropy A_B_.

#### 3.4.3. Debye Temperature

We have calculated the Debye temperature (ϴ_ϴ_) from the average sound velocity (*V_m_*) using the equation [36]:

(16) θD=hkB34π13νm

where *h* and *k_B_* are the Planck’s and Boltzmann’s constants, respectively, and V_a_ is the atomic volume. The average sound velocity in polycrystalline systems, $\nu_{m}$, is evaluated by the expression:

(17) $$\nu_{m}={\left[\frac{1}{3}\left(\frac{2}{\nu_{t}^{3}}+\frac{1}{\nu_{l}^{3}}\right)\right]}^{-1/3}$$

where $\nu_{t}$ and $\nu_{l}$ are the mean longitudinal and transverse sound velocities, which can be related by the shear and bulk moduli from Navier’s equations:

(18) $$\nu_{t}={\left(\frac{3B+4G}{3\rho }\right)}^{1/2}\;\;and\;\;\nu_{t}={\left(\frac{G}{\rho }\right)}^{1/2}.$$

Table 6 shows the calculated volumetric density, sound velocities, and Debye temperature as a function of Na removal. Derived from the calculated elastic constants, the calculated Debye temperature $\theta_{D}$ for Na_1_MnPO_4_ was found to be 512.7 K. Unfortunately, we were unable to uncover any experimental or theoretical data with which to compare our computed results. As seen in Table 5, Na deintercalation resulted in a monotonic drop in all sound velocities as well as the $\theta_{D}$ for all the systems. Considering that the $\theta_{D}$ in a solid may be used to define its covalent strength, the drop in $\theta_{D}$ as a results of Na deintercalation indicated that the covalent strength of NaMnPO_4_ decreases with each deintercalation step. Although the thermal conductivity of the material increases with increasing $\theta_{D}$ in general, our results indicated a modest drop in $\theta_{D}$, indicating that the thermal conductivity of the material was maintained after Na deintercalation. It is noteworthy that the volumetric density, sound velocities, and Debye temperature increase during the fourth step of Na deintercalation, with a Debye temperature calculated to be 497.8 K. 

**Figure 5:**
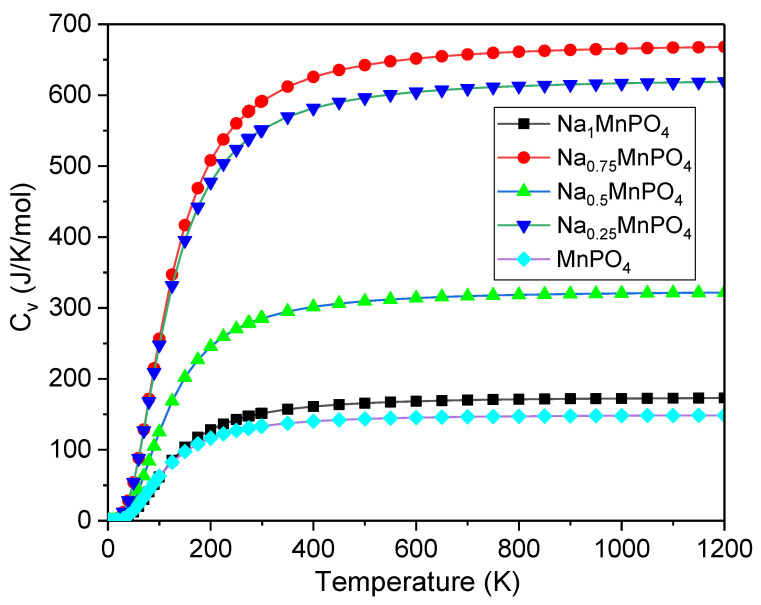
Temperature specific heat at constant volume (*C_v_*) of Na_x_MnPO_4_ (x = 1, 0.75, 0.5, 0.25, 0).

**Figure 6:**
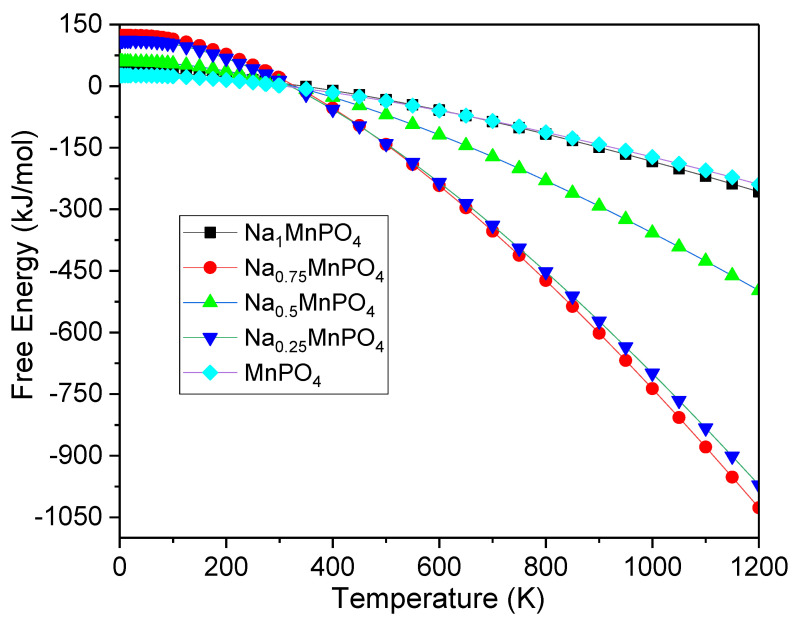
The free energy versus temperature for Na_x_MnPO_4_ (x = 1, 0.75, 0.5, 0.25, 0).

**Figure 7:**
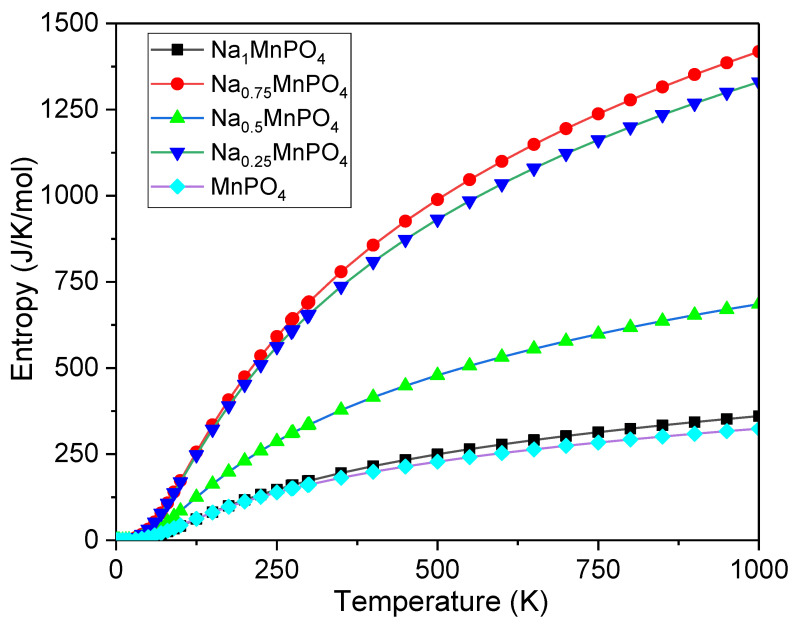
Entropy vs. temperature for Na_x_MnPO_4_ (x = 1, 0.75, 0.5, 0.25, 0).

Using the MedeA VASP program, certain essential thermodynamic parameters such as specific heat at constant volume (C_v_), free energy, and entropy are calculated at 0 GPa pressure and temperatures (0–1200 K) to investigate thermodynamic properties. From Figure 5, Figure 6 and Figure 7, the influence of temperature on C_v_, free energy, and entropy has been calculated and shown to be significant. At low temperatures, the rate of increase in C_v_ is rapid for all intercalation phases, regardless of the intercalation stage. During high-temperature intercalation, the value of C_v_ of all intercalation stages approaches the classical asymptotic limit, which is also known as the Dulong–Petit limit [37]. At low temperatures, the fluctuation of free energy versus temperature, as well as the variation of C_v_ versus temperature, shows that the increase in free energy is significant for all intercalation stages. Unfortunately, no experimental data or other theoretical conclusions are available to compare. We hope that our research will be useful in future experiments.

## 4. Conclusions

The effect of Na atom deintercalation on the structural, electronic, mechanical, and thermodynamic properties of NaMnPO_4_ has been investigated by first-principle calculations. The calculated lattice constants show good agreement with the experimental values to within 5%. During the Na deintercalation stages, the lattice parameters and the volume showed a deviation of less than 6%, which is not enough to cause irreversible distraction to the system. Electronic DOS revealed that during the Na removal stages, between the 1st and 3rd stages, the material showed an increase in metallicity, while on the other hand during the 4th stage, the material showed a semiconductor behavior with a band gap of 0.194 eV. The voltage window of 3.997 to 3.848 V was obtained, and the calculated formation energy values were found to be negative, which symbolizes the prediction of a solid possessed by the material. The calculated elastic constants suggested mechanical stability for NaMnPO_4_ since the stability criteria were satisfied for all deintercalated systems except fully deintercalated MnPO_4_. According to the Pugh criterion of ductility and brittleness, we note that the Na_1_MnPO_4_, Na_0_._75_MnPO_4_, and MnPO_4_ structures are ductile, while Na_0_._5_MnPO_4_ and Na_0_._25_MnPO_4_ are both brittle.

## Figures and Tables

**Table 1:** Calculated lattice parameters of NaMnOP_4_ and previously reported experimental results.

Lattice Parameter	PBE + U = 5	Exp [25]	% Deviations
a (Å)	10.693	10.528	1.555
b (Å)	6.421	6.321	1.570
c (Å)	5.053	4.985	1.355
Volume (Å^3^)	346.995	331.74	4.951

**Table 2:** Calculated lattice parameters of deintercalated NaxMnOP_4_.

	a (Å)	b (Å)	c (Å)	V (Å^3^)	V Deviation (%)	a Deviation (%)	b Deviation (%)	c Deviation (%)
Na_1_MnPO_4_	10.693	6.421	5.053	346.995	––	––	––	––
Na_0.75_MnPO_4_	10.537	6.325	5.066	337.644	2.7	1.5	1.5	0.3
Na_0.5_MnPO_4_	10.350	6.231	5.062	326.453	3.3	1.8	1.5	0.1
Na_0.25_MnPO_4_	10.082	6.166	5.028	312.509	4.3	2.6	1.0	0.7
MnPO_4_	9.874	5.057	4.918	294.152	5.9	2.1	1.8	2.2

**Table 3:** Elastic constants (C_ij_) for Na_x_MnPO_4_ olivine polymorphs (x = 1, 0.75, 0.5, 0.25, 0).

C_ij_	Na_1_MnPO_4_	Na_0.75_MnPO_4_	Na_0.5_MnPO_4_	Na_0.25_MnPO_4_	MnPO_4_
C_11_	124.46	116.46	119.25	116.19	201.63
C_12_	61.43	50.10	35.94	17.73	55.84
C_13_	58.55	48.93	43.67	36.24	72.41
C_22_	125.29	127.87	140.41	171.12	153.41
C_23_	51.49	33.59	21.92	9.29	38.30
C_33_	148.46	131.67	114.56	94.66	139.66
C_44_	42.28	32.12	25.48	24.25	−39.20
C_55_	49.01	47.22	46.74	45.05	56.59
C_66_	46.71	38.99	36.35	33.95	11.88
C_11_ + C_22_ − 2C_12_	126.89	144.13	187.78	251.85	243.36
C_11_ + C_33_ − 2C_13_	155.82	150.27	146.47	138.37	196.47
C_22_ + C_33_ − 2C_23_	170.79	192.36	211.13	247.20	216.47

**Table 4:** Elastic constants (C_ij_) for Na_x_MnPO_4_ olivine polymorphs (x = 1, 0.75, 0.5, 0.25, 0).

	B_H_	G_H_	E_H_	B/G	*ν*
Na_1_MnPO_4_	81.48	42.36	108.3	1.924	0.2785
Na_0.75_MnPO_4_	69.97	38.78	98.19	1.805	0.2661
Na_0.5_MnPO_4_	63.08	38.34	95.63	1.655	0.2473
Na_0.25_MnPO_4_	53.76	37.25	90.74	1.443	0.2185
MnPO_4_	89.81	35.73	94.05	2.514	0.1384

**Table 5:** Anisotropy in the shear elastic factor (A_i_ with *i* = 1,2,3) and anisotropy in compressibility and shear moduli (A_B_ and A_G_ in %).

	$A_{1}$	$A_{2}$	$A_{3}$	$A_{B}$	$A_{G}$
Na_1_MnPO_4_	1.085	1.145	1.409	1.4	1.2
Na_0.75_MnPO_4_	0.855	0.989	1.065	1.9	1.3
Na_0.5_MnPO_4_	0.696	0.886	0.844	4.1	3.7
Na_0.25_MnPO_4_	0.701	0.937	0.632	10.9	9.8
MnPO_4_	−0.798	1.046	0.226	8.9	7.6

**Table 6:** Calculated volumetric density ρ (in kg/m^3^), longitudinal $\nu_{l}$ transverse $\nu_{t}$, and average sound velocities $\nu_{t}$ in m/s, and Debye temperature $\theta_{D}$ in Kelvin.

	ρ	$\nu_{t}$	$\nu_{l}$	$\nu_{m}$	$\theta_{D}$
Na_1_MnPO_4_	3309	3576	6454	3984	512.7
Na_0.75_MnPO_4_	3288	3435	6084	3821	490.0
Na_0.5_MnPO_4_	3284	3417	5897	3792	485.0
Na_0.25_MnPO_4_	3308	3357	5593	3713	476.2
MnPO_4_	3385	3379	5216	3707	497.8

## Data Availability

Not applicable.
